# Metabolic Activation of Benzo[*a*]pyrene by Human Tissue Organoid Cultures

**DOI:** 10.3390/ijms24010606

**Published:** 2022-12-29

**Authors:** Angela L. Caipa Garcia, Jill E. Kucab, Halh Al-Serori, Rebekah S. S. Beck, Franziska Fischer, Matthias Hufnagel, Andrea Hartwig, Andrew Floeder, Silvia Balbo, Hayley Francies, Mathew Garnett, Meritxell Huch, Jarno Drost, Matthias Zilbauer, Volker M. Arlt, David H. Phillips

**Affiliations:** 1 Department of Analytical, Environmental and Forensic Sciences, School of Cancer & Pharmaceutical Sciences, King’s College London, London SE1 9NH, UK; 2 Department of Food Chemistry and Toxicology, Karlsruhe Institute of Technology, Institute of Applied Biosciences, 76131 Karlsruhe, Germany; 3 Division of Environmental Health Sciences, School of Public Health and Masonic Cancer Center, University of Minnesota, Minneapolis, MN 55455, USA; 4 Wellcome Sanger Institute, Cambridge CB10 1SA, UK; 5 Max Planck Institute of Molecular Cell Biology and Genetics, 01307 Dresden, Germany; 6 Princess Máxima Center for Pediatric Oncology, Oncode Institute, 3584 CS Utrecht, The Netherlands; 7 Department of Paediatrics, University of Cambridge, Cambridge CB2 0QQ, UK

**Keywords:** carcinogen, 3D culture, benzo[*a*]pyrene, human tissue organoid, CYP1A1, NQO1, DNA adducts, RT-qPCR, DNA damage response

## Abstract

Organoids are 3D cultures that to some extent reproduce the structure, composition and function of the mammalian tissues from which they derive, thereby creating in vitro systems with more in vivo-like characteristics than 2D monocultures. Here, the ability of human organoids derived from normal gastric, pancreas, liver, colon and kidney tissues to metabolise the environmental carcinogen benzo[*a*]pyrene (BaP) was investigated. While organoids from the different tissues showed varied cytotoxic responses to BaP, with gastric and colon organoids being the most susceptible, the xenobiotic-metabolising enzyme (XME) genes, *CYP1A1* and *NQO1*, were highly upregulated in all organoid types, with kidney organoids having the highest levels. Furthermore, the presence of two key metabolites, BaP-*t*-7,8-dihydrodiol and BaP-tetrol-l-1, was detected in all organoid types, confirming their ability to metabolise BaP. BaP bioactivation was confirmed both by the activation of the DNA damage response pathway (induction of p-p53, pCHK2, p21 and γ-H2AX) and by DNA adduct formation. Overall, pancreatic and undifferentiated liver organoids formed the highest levels of DNA adducts. Colon organoids had the lowest responses in DNA adduct and metabolite formation, as well as XME expression. Additionally, high-throughput RT-qPCR explored differences in gene expression between organoid types after BaP treatment. The results demonstrate the potential usefulness of organoids for studying environmental carcinogenesis and genetic toxicology.

## 1. Introduction

Benzo[*a*]pyrene (BaP) is a well-characterised human carcinogen that is ubiquitous in the environment, present in polluted air (including tobacco smoke), water, soil and food [1]. BaP is a polycyclic aromatic hydrocarbon (PAH), a class of compounds formed by the incomplete combustion of organic matter, and it is often used as representative of the biological effects of PAHs [2,3]. It induces tumour formation at multiple sites in experimental animals, including in the lung, liver, forestomach, skin, lymphoid tissues and sarcomas in mice; lung, mammary glands and sarcomas in rats; and lung, trachea, larynx, forestomach and sarcomas in hamsters [1]. Environmental and occupational exposure to mixtures of PAHs containing BaP is widely associated with increased risk of cancer in humans, including lung and skin cancer [1].

BaP requires metabolic activation to exert its biological effects, including genotoxicity. It is mainly catalysed by cytochrome P450 (CYP) 1A1 and CYP1B1 [4] and epoxide hydrolase to produce the ultimate reactive species BaP-7,8-diol-9,10-epoxide (BPDE) which forms bulky DNA adducts preferentially at the *N*^2^ position of guanines, resulting in 10-(deoxyguanosin-*N*^2^-yl)-7,8,9-trihydroxy-7,8,9,10-tetrahydro-BaP (dG-*N*^2^-BPDE) [4,5]. These DNA adducts have been reported in many mammalian cell lines and animal tissues following BaP exposure, including in lung, liver, colon, forestomach, glandular stomach, kidney, pancreas, and spleen [6,7,8,9,10,11] and in human lung [12].

DNA adduct formation is considered an initiation event of carcinogenesis, although additional events are required for tumour formation. Some of these changes have been investigated in mice and human cell lines and include differential expression of several genes and miRNAs, as well as epigenetic alterations [6,13,14,15,16]. These studies help understand tissue-specific responses to BaP but may not all be relevant to humans. Recently, 3-dimensional (3D) cellular monocultures of primary human bronchial epithelial cells, HepG2 cell spheroids and organ-on-chip technologies have examined the toxicity of BaP and other PAHs, identifying chemical-specific transcriptional patterns, and showing differences in sensitivity between 2D and 3D models [13,17,18]. Organoids, which are multicellular 3D cultures derived from stem cells that self-assemble into structures that contain organ-specific cell types, have been shown to recreate some of the in vivo cell architecture and functions of the organ of origin [19,20,21,22,23]. They are thus more representative of human physiology and may be helpful in assessing biologically relevant effects of environmental carcinogens. Although some studies have used human organoids to study the effects of environmental agents (reviewed in [24]), they have not yet been utilised to study the effects of BaP.

Here, normal human tissue organoids from stomach, pancreas, liver, colon, and kidney were treated with BaP. The metabolic competence of the organoids was evaluated by examining the formation of BaP metabolites and DNA adducts (i.e., dG-*N*^2^-BPDE). Tissue-specific responses were evaluated by induction of DNA damage response (DDR) proteins and mRNA expression changes of genes involved in different pathways including DDR, apoptosis and xenobiotic metabolism.

## 2. Results

### 2.1. Cell Viability of Human Tissue Organoids

**Figure 1:**
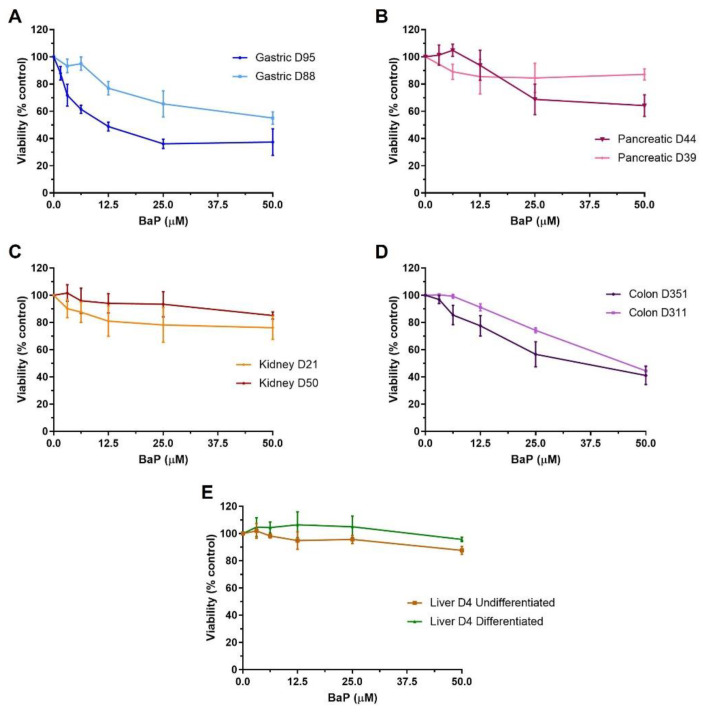
Cell viability in human tissue organoids after BaP treatment. Organoids from normal human stomach (**A**) D95 and D88, pancreas (**B**) D39 and D44, kidney (**C**) D50 and D21, colon (**D**) D351 and D311, and liver (**E**) D4 undifferentiated and differentiated tissues were treated with various BaP concentrations (0–50 µM) for 48 h. Vehicle controls (0.5% DMSO) were included. Cell viability (% control) was measured using the CellTiter-Glo assay. Results are shown as mean ± SEM (n ≥ 3).

Differences in viability were seen between different tissues exposed to BaP (0–50 µM; 48 h) and in some cases between donor cultures (n = 2 for gastric, liver, colon and kidney; n = 1 for pancreas), with gastric and colon organoids being the most susceptible to BaP cytotoxicity. Gastric organoids from donor culture D95 were more susceptible than those from donor culture D88, IC_50_ 12.8 µM versus close to 50 µM (Figure 1A). Colon organoids had IC_50_ values of 44.2 µM and 34.5 µM for D311 and D351, respectively (Figure 1D). Pancreatic, kidney and liver organoids had IC_50_ values greater than 50 µM. Pancreatic culture D39 was less susceptible to BaP-induced cytotoxicity than D44 (Figure 1B), and kidney culture D21 was slightly more susceptible than D50 (Figure 1C). No cytotoxicity was seen in either undifferentiated or differentiated liver organoids from donor D4 (Figure 1E). Based on these results, concentrations that induced 20–40% and 40–60% cell viability were chosen for subsequent experiments with each organoid culture. When there was no clear IC_50_ or it was close to the highest concentration, 50 µM and a lower concentration (12.5 µM) were selected.

### 2.2. Xenobiotic-Metabolising Enzyme (XMEs) Expression

**Figure 2:**
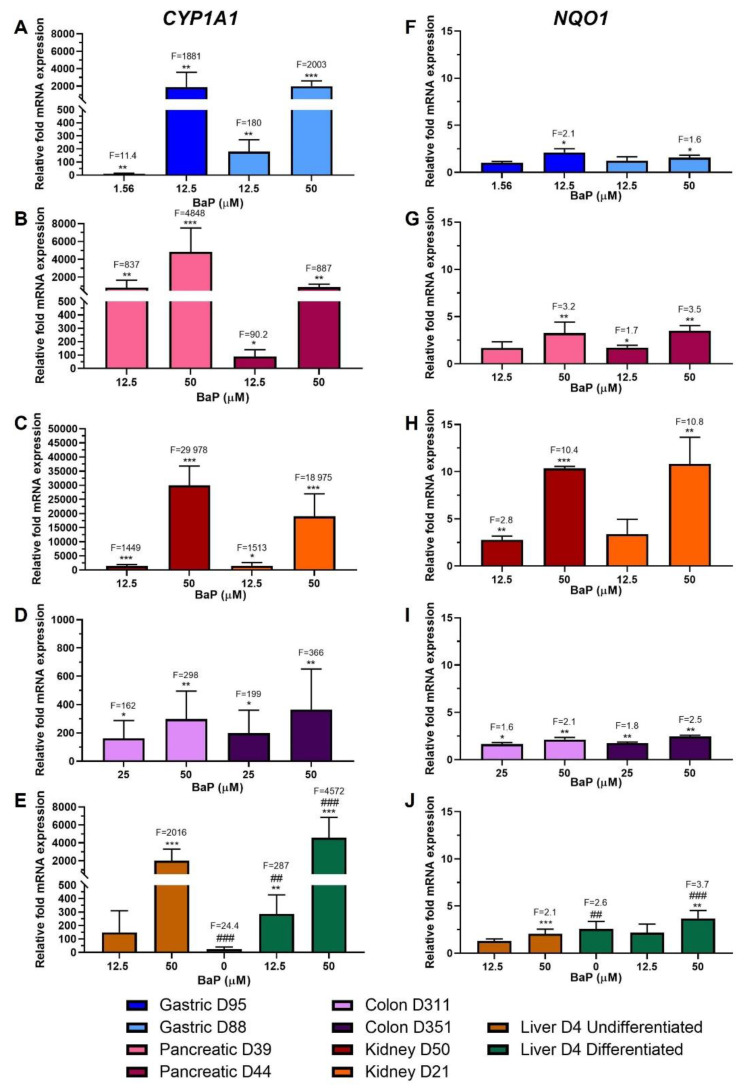
Relative gene expression of XMEs in human tissue organoids after BaP treatment. RT-qPCR and the 2^−ΔΔCT^ method were used to determine CYP1A1 and NQO1 expression in gastric (D95 and D88; (**A**,**F**)), pancreatic (D39 and D44; (**B**,**G**)), kidney (D50 and D21; (**C**,**H**), colon (D311 and D351; (**D**,**I**)) and liver undifferentiated and differentiated (D4; (**E**,**J**)) organoids treated with the indicated BaP concentrations for 48 h. Values were normalised to mRNA expression of the housekeeping gene GAPDH and are relative to the vehicle control (0.5% DMSO); for liver organoids the values are relative to the undifferentiated control. Results are shown as mean ± SD (n ≥ 3). Statistical analysis was performed by log2 transforming the data and a one sample t-test with Bonferroni correction against the control mean of 0: * *p* < 0.05; ** *p* < 0.01; *** *p* < 0.001 compared to untreated control; ^##^ *p* < 0.01; ^###^ *p* < 0.001 compared to undifferentiated liver control.

To assess the ability of the organoids to metabolically activate BaP, gene expression levels of two of the main XMEs involved in BaP metabolism, CYP1A1 and NQO1, were investigated by RT-qPCR. In general, there was a concentration-dependent upregulation of CYP1A1 and NQO1 in all organoid donor cultures (Figure 2A–J).

CYP1A1 upregulation by BaP was significant in both gastric organoid cultures, with an almost 2000-fold increase at the highest concentrations, and 11.4-fold and 180-fold increases for D95 and D88, respectively, at the lowest concentrations (Figure 2A). Pancreatic donor culture D39 had much higher levels than D44 at both concentrations, with increases of almost 5000-fold at 50 µM and around 800-fold at 12.5 µM for D39, and around 900-fold at 50 µM and 90-fold at 12.5 µM for D44 (Figure 2B). Expression levels of CYP1A1 were highest in kidney organoids, having inductions of almost 30,000- and 19,000-fold in D50 and D21, respectively, at 50 µM and of ~1500-fold at 12.5 µM (Figure 2C). Colon organoids showed the lowest CYP1A1 induction levels overall, with increases of between 300- and 400-fold at the higher concentration in D311 and D351, respectively, and around 200-fold at the lower (Figure 2D). CYP1A1 levels in differentiated liver D4 prior to treatment were significantly higher (24-fold) than those in undifferentiated D4. After treatment, induction at 50 µM in differentiated liver organoids was around twice that in undifferentiated (~4500- and 2000-fold, respectively), relative to undifferentiated liver control. At 12.5 µM, induction was significant only in differentiated organoids, with a 287-fold change compared to undifferentiated control. Induction of CYP1A1 was also significant at both concentrations compared to differentiated control (Figure 2E).

In gastric organoids, significant changes in NQO1 expression were seen only at the highest concentrations with fold increases of 2.1 and 1.6 for D95 and D88, respectively (Figure 2F). Both pancreatic organoid cultures had very similar induction levels; at 50 µM they had approximately a 3.5-fold increase, while at 12.5 µM only D44 had a significant induction of 1.7-fold (Figure 2G). As with CYP1A1, higher induction levels were seen in kidney organoids, with significant increases in expression levels of around 10-fold at 50 µM in both donor cultures; however, at 12.5 µM induction was only significant in D50 with a 2.8-fold increase (Figure 2H). NQO1 expression increased significantly for both colon organoid donor cultures, with around 1.5- and 2.5-fold at 25 µM and 50 µM, respectively (Figure 2I). Prior to treatment, differentiated liver organoids had significantly higher levels of NQO1 (2.6-fold) than undifferentiated organoids. Induction was significant only at 50 µM BaP, with a 2.1-fold increase for undifferentiated organoids and a 3.7-fold increase for differentiated organoids compared to undifferentiated liver control. Differentiated organoids also had a significant induction at 50 µM relative to differentiated control (Figure 2J).

### 2.3. DDR Protein Induction

**Figure 3:**
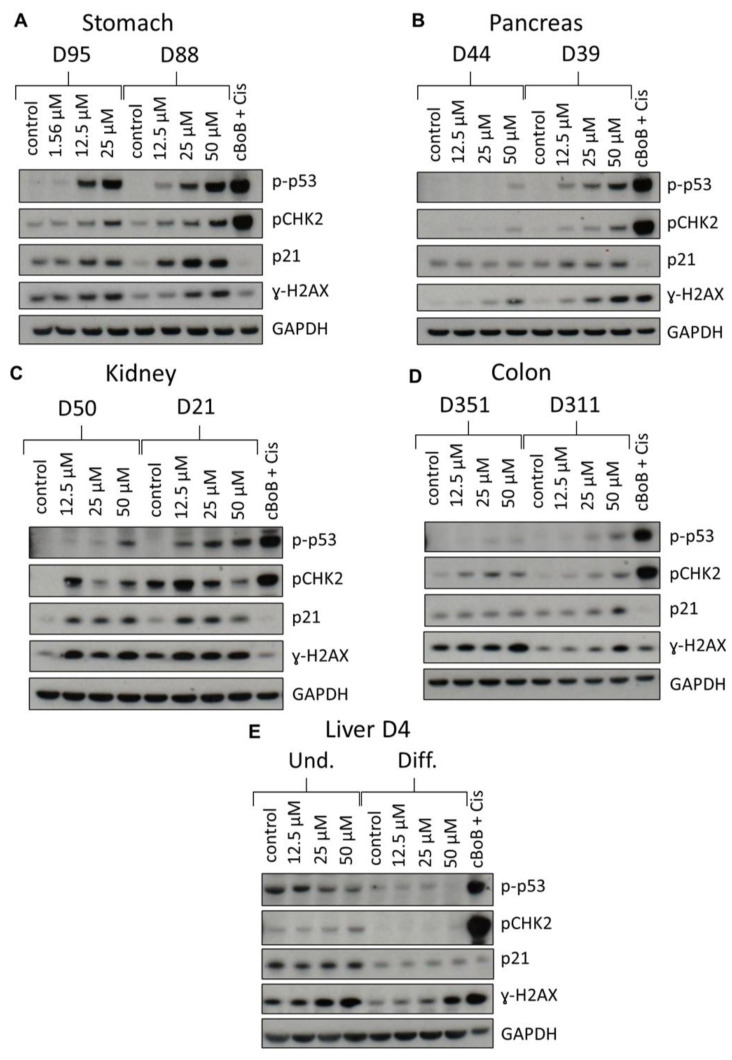
DDR in normal human tissue organoids after BaP treatment. Organoids from gastric D95 and D88; (**A**), pancreatic D39 and D44; (**B**), kidney D50 and D21; (**C**), colon D351 and D311; (**D**) and liver (D4 undifferentiated and differentiated; (**E**) tissues were treated with the indicated BaP concentrations for 48 h, and lysates were analysed by Western blotting. Various DDR proteins (p-p53, pCHK2, p21 and γ-H2AX) were detected and GAPDH was used as a loading control. cBoB + Cis (cBoB treated with 3.125 μM cisplatin) was used as a positive control. Representative blots are shown (n = 2).

To further evaluate BaP effects in organoids, induction of DDR proteins (p-p53, pCHK2, p21 and γ-H2AX) was investigated by Western blotting. An additional concentration causing 60–80% viability, or 25 µM when there was no IC_50_, was also examined. All DDR proteins were induced in both gastric cultures (Figure 3A). However, pCHK2 and p21 expression was higher in D88 than in D95. Induction of p-p53, pCHK2 and p21 was concentration-dependent in both cultures, while induction of γ-H2AX was concentration-dependent only in D88.

Although all proteins were induced in pancreatic organoids, culture D44 had very low levels of expression, and no induction in p21, compared to D39 (Figure 3B). Culture D39 showed a concentration-dependent induction in all DDR proteins except p21, where the level of induction remained constant at all BaP concentrations.

Kidney organoids showed expression and induction of all DDR proteins (Figure 3C). Expression levels of p-p53 were higher in D21 than in D50 and induction was concentration dependent, whilst pCHK2 showed the highest induction at 12.5 μM, which then decreased at the highest concentrations tested. p21 was induced at all concentrations, but to a similar extent in D50, while in D21 induction was slightly decreased at the highest concentration tested. There was γ-H2AX induction in both donor cultures, but the induction was constant across treatments (Figure 3C).

Most DDR proteins were expressed in colon organoids, but induction was seen only for pCHK2 and γ-H2AX in both donor cultures, and for p-p53 and p21 in D311 (Figure 3D).

Undifferentiated liver organoids had higher expression levels of all DDR proteins than differentiated organoids. Undifferentiated liver organoids showed induction of pCHK2 and γ-H2AX, while p-p53 levels seemed to decrease. Differentiated organoids had low expression of all DDR proteins and only γ-H2AX was induced (Figure 3E).

### 2.4. HPLC Fluorescence Analysis of BaP Metabolites

**Figure 4:**
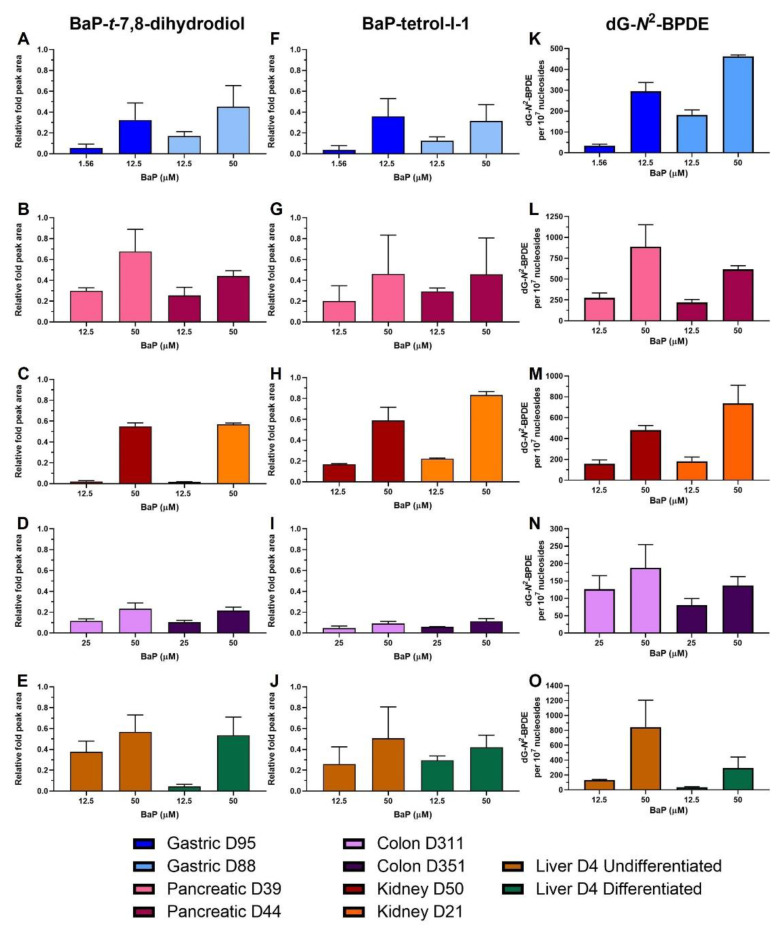
BaP metabolite and DNA adduct levels in human tissue organoids after BaP treatment. Gastric D95 and D88; (**A**,**F**,**K**), pancreatic D39 and D44; (**B**,**G**,**L**), kidney D50 and D21; (**C**,**H**,**M**), colon (D311 and D351; (**D**,**I**,**N**) and liver undifferentiated and differentiated (D4; (**E**,**J**,**O**) organoids were treated with the indicated BaP concentrations for 48 h. Vehicle controls (0.5% DMSO) were included (not shown). The formation of BaP-t-7,8-dihydrodiol (**A**–**E**) and BaP-tetrol-l-1 (**F**–**J**) was determined by HPLC analysis. Metabolite levels are presented as peak area relative to phenacetine (arbitrary units). dG-*N*^2^-BPDE adduct formation was quantified using LC-ESI-MS/MS (**K**–**O**). Results are shown as mean ± SD (n ≥ 3).

In order to assess the capability of the organoids to metabolise BaP, the levels of two major metabolites, BaP-t-7,8-dihydrodiol (diol; precursor of BPDE) and BaP-tetrol-l-1 (tetrol; hydrolysis product of BPDE) were measured. They were formed by all organoid types in a dose-dependent manner (Figure 4A–J). Similar levels of both metabolites were detected in gastric organoids, with slightly higher levels of diol in D88 and of tetrol in D95 (Figure 4A,F). Pancreatic D39 organoids had higher diol levels at the highest concentration tested (Figure 4B,G). In kidney organoids similar levels of diol were detected in both D50 and D21 donor cultures, and slightly higher levels of tetrol in D21 (Figure 4C,H). Colon cultures had very similar levels of both metabolites (Figure 4D,I). While the metabolites formed in both undifferentiated and differentiated liver organoids, diol levels at 12.5 µM were higher in undifferentiated liver (Figure 4E,J).

### 2.5. BaP-DNA Adduct Formation

Overall, dG-*N*^2^-BPDE in organoid DNA was formed in a concentration-dependent manner and levels varied between organoid types and donor cultures (Figure 4K–O). Gastric D95 had lower adduct levels than gastric D88 at IC_50_ values, with D95 having 295 adducts per 10^7^ nucleosides (12.5 µM) and D88 formed 463 adducts per 10^7^ nucleosides (50 µM). Similarly, at the lower concentrations culture D95 had 34.1 adducts per 10^7^ nucleosides while D88 had 182 adducts per 10^7^ nucleosides; however, the concentration used to treat D88 was slightly more cytotoxic than that for D95 (Figure 4K). Pancreatic organoids had higher adduct levels, with D39 having the highest with 889 and 275 adducts per 10^7^ nucleosides at 50 and 12.5 µM, respectively. Culture D44 had 617 adducts per 10^7^ nucleosides at 50 µM and 220 adducts per 10^7^ nucleosides at 12.5 µM (Figure 4L). Kidney D21 had higher adduct levels than D50; however, the concentrations tested were also slightly more cytotoxic in D21 than in D50. Culture D21 had 182 adducts per 10^7^ nucleosides at 12.5 µM and 738 adducts per 10^7^ nucleosides at 50 µM, while D50 had 159 and 481 adducts per 10^7^ nucleosides at 12.5 and 50 µM, respectively (Figure 4M). Colon organoids had the lowest adduct levels of all the organoids with D351 having lower levels (137 adducts per 10^7^ nucleosides) than D311 (188 adducts per 10^7^ nucleosides) after 50 µM BaP. At 25 µM, D351 and D311 had 81 and 126 adducts per 10^7^ nucleosides, respectively, although this concentration was more cytotoxic in D351 (Figure 4N). Lastly, undifferentiated liver organoids had much higher dG-*N*^2^-BPDE levels than differentiated liver organoids (131 vs. 37 adducts per 10^7^ nucleosides at 12.5 µM; 844 vs. 295 at 50 µM) (Figure 4O).

### 2.6. Gene Expression Changes

Genes were selected for HT RT-qPCR on the basis of their involvement in genomic instability, DNA damage response and repair, oxidative stress response, apoptosis, cell cycle arrest and proliferation, and xenobiotic metabolism [25]. Genes with log2-fold values higher than 1 (expression doubled) or lower than −1 (expression halved) after BaP treatment compared to control were considered biologically relevant. Overall, several differences in gene expression were seen between organoid types and donor cultures. Pancreas, kidney, and colon organoids had the most relevant changes, and although gene expression between donor cultures of these tissues appeared consistent, slight differences were seen in some cases. Changes between gastric donor cultures, and undifferentiated and differentiated liver organoids had more notable differences, as gastric organoid D88 and undifferentiated liver had more relevant changes compared to gastric organoid D95 and differentiated liver. Concentration-dependent differences were also seen, with the highest concentration having more pronounced changes for some genes (Supplementary Figure S1). Supplementary Table S3 shows the genes that had biologically relevant changes in at least three organoid types.

**Figure 5:**
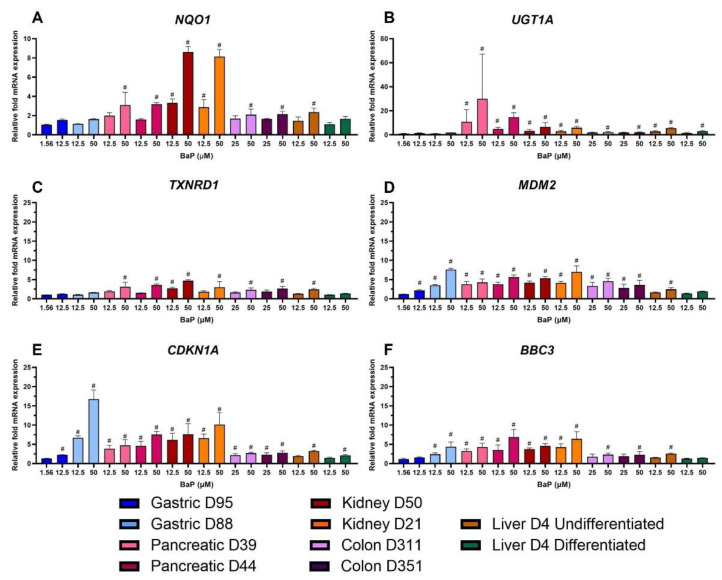
Effects of BaP on gene expression related to xenobiotic metabolism, oxidative stress response, transcription, proliferation and cell cycle control, and apoptosis. Human tissue organoids were treated with the indicated BaP concentrations for 48 h. Gene expression changes were measured by HT RT-qPCR. Linear fold-changes for (**A**) NQO1, (**B**) UGT1A, (**C**) TXNRD1, (**D**) MDM2, (**E**) CDKN1A and (**F**) BBC3 are shown as mean ± SD (n = 3). Log2 values ±1 were considered biologically relevant (#), compared to the vehicle control (0.5% DMSO).

The most significant changes in the genes involved in xenobiotic metabolism (XM) were seen in NQO1 and UGT1A. NQO1 showed similar levels of expression in all organoid types except kidney, which had higher fold-change values. However, the results were only biologically relevant for pancreas, colon and undifferentiated liver at the highest concentrations, and for kidney at both concentrations (Figure 5A). UGT1A, involved in BaP detoxication [26], was upregulated in all organoids; however, pancreatic organoids had the highest expression followed by kidney organoids. The lowest fold changes for UGT1A were seen in gastric organoids, which were not biologically significant (Figure 5B). The expression of TXNRD1, which is part of the oxidative stress response (OS) group, increased in all organoids; however, this was only biologically significant in pancreas, colon, undifferentiated liver, and kidney organoids (Figure 5C). MDM2, a major regulator of the p53 pathway, was selected as the gene with the most biologically relevant changes in the transcription factor (TF) group. It was upregulated and had significant changes in all organoids except the differentiated liver (Figure 5D). CDKN1A, which encodes for the cyclin-dependent kinase inhibitor p21, was the gene in the proliferation and cell cycle control (PCC) group with most biologically relevant changes. It was upregulated in all organoids, but its levels of expression were much higher in gastric D88 than in other organoids. The lowest levels were seen in gastric D95, and in colon and liver organoids (Figure 5E). The pro-apoptotic gene BBC3 showed greater expression changes in kidney, pancreatic and gastric D88 organoids; upregulation was not significant in gastric D95 and differentiated liver organoids (Figure 5F).

**Figure 6:**
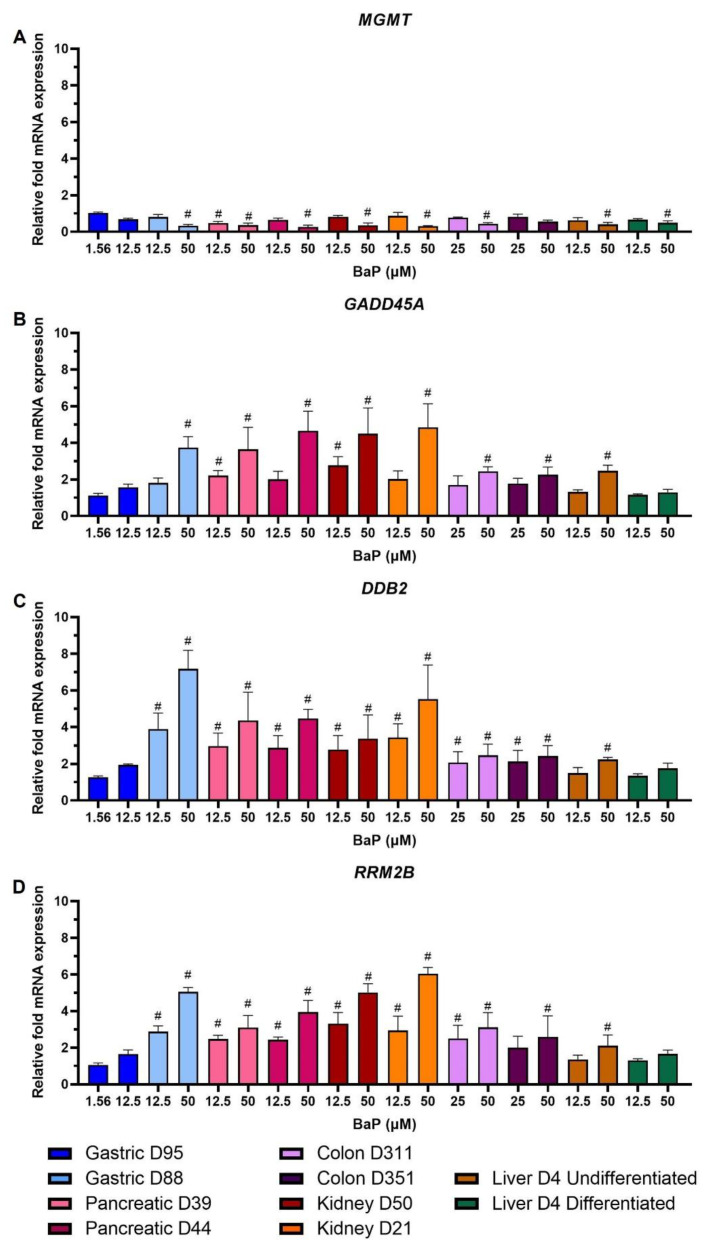
Effects of BaP on gene expression related to DNA damage response and repair. Human tissue organoids were treated with the indicated BaP concentrations for 48 h. Gene expression changes were measured by HT RT-qPCR. Linear fold-changes for (**A**) MGMT, (**B**) GADD45A, (**C**) DDB2 and (**D**) RRM2B are shown as mean ± SD (n = 3). Log2 values ±1 were considered biologically relevant (#), compared to the vehicle control (0.5% DMSO).

Of the genes with the most biologically relevant changes from the DDR group, MGMT expression was downregulated in all organoids in a concentration-dependent manner (Figure 6A). GADD45A was upregulated concentration-dependently across all organoids, with highest levels in kidney, pancreas and gastric D88 organoids (Figure 6B). For both MGMT and GADD45A, expression changes were not significant for gastric D95 and at low concentrations for most other donor cultures. For DDB2 and RRM2B, upregulation was biologically relevant in almost all cultures and conditions, except for gastric D95 and differentiated liver at both concentrations, and at the low concentration in undifferentiated liver, and colon D351 for RRM2B only (Figure 6C,D).

## 3. Discussion

This study explored the ability of different human tissue organoids to bioactivate BaP. The organoids were derived from normal human tissues and consisted of organ-specific epithelial cells and stem cells. Gastric organoids mainly consisted of gland mucous and chief cells [19]. Colon organoids consisted of intestinal epithelium purified from sigmoid colon fragments [23]. Kidney organoids, also known as ‘tubuloids’, consist of tubular epithelial cells that expressed proximal and distal tubule markers, as well as collecting duct and loop of Henle markers [22]. Pancreatic organoids consisted of ductal cells [20]. Lastly, undifferentiated liver organoids consist of bile duct cells, additionally, these were differentiated into hepatocytes that carry out most of the xenobiotic metabolism in this organ [21].

Assessment of cell viability after BaP treatment showed that gastric D95 cultures were most susceptible to BaP cytotoxicity with an IC_50_ concentration around 4-fold lower (i.e., 12.8 µM) than gastric D88, pancreatic, kidney and liver organoids (i.e., ≤50 µM), and around 3-fold lower than colon organoids (i.e., 44.2 and 34.5 µM) (Figure 1). Although treatment conditions differ, only low cytotoxic responses have been reported after BaP treatment in several cell lines, including human colon HCT116, human hepatocellular carcinoma HepG2 and human breast adenocarcinoma MCF-7 and MDA-MB-231 cells [18,27,28,29,30]. Similarly, treatment of human bronchial epithelial cells cultured in an air–liquid interface did not affect cell viability [13]. In addition, others reported high toxicity in HepG2 cells and low toxicity in the kidney HEK293 cells under static conditions; however, effects on cell viability seemed marginal in a liver–kidney organ-on-chip [17]. Furthermore, high BaP cytotoxicity in HepG2 cells and human lymphoblastoid MCL-5 cells was reported in four different human cell lines [31].

The potential of the organoids to metabolically activate BaP was evaluated by examining gene expression levels of *CYP1A1* and *NQO1*. Overall, both were upregulated in a concentration-dependent manner in all organoid types and donor cultures. Highest levels were seen in kidney organoids and lowest in colon organoids (Figure 2). Induction of mRNA expression of these enzymes after BaP treatment has been reported in different experimental models, including mice and rats as well as cultured human and animal cells [6,13,18,27,30,31,32,33,34]. This upregulation correlated with increased CYP1A1 activity in human cell lines [31,35] and increased NQO1 activity in mice after treatment [33].

Induction of XME gene expression indicated that organoids are capable of bioactivating BaP. To corroborate this further, expression of DDR proteins was investigated, showing activation of DDR pathways in all organoid types by BaP (Figure 3). Although levels differed, concentration-dependent responses were seen in p-p53 expression in both gastric and kidney cultures, pancreatic D39 and colon D311, while pancreatic culture D44 only showed induction at the highest concentration. In colon D351 and liver undifferentiated organoids no induction was found. Interestingly, undifferentiated liver organoids showed a reduction of p-p53 expression, which could be due to cell death unrelated to BaP toxicity as these were cultured for a longer period due to the differentiation protocol. Induction of the p53 downstream target p21 was also observed in gastric and kidney organoids, pancreatic D39 and colon D311 organoids. Induction of p-CHK2 in all organoids, except differentiated liver organoids, further suggests activation of the ATM pathway. This is supported by previous reports showing the induction and activation of p53, p21 and CHK2 by BaP and its reactive metabolite BPDE in human and murine cells [30,32,34,36,37,38]. Expression of γ-H2AX increased after BaP treatment in all organoids, in line with the induction seen in human and murine cell lines as well as in human primary and stem cells [36,37,38,39].

Moreover, BaP activation was confirmed by the concentration-dependent formation of BaP-*t*-7,8-dihydrodiol and BaP-tetrol-l-1 (Figure 4A–J) with metabolite levels varying between organoid types. Many studies have evaluated BaP metabolism in different experimental systems identifying different amounts of these and other BaP metabolites. Studies using hepatic microsomes from BaP-pretreated wild-type, Hepatic Reductase Null (HRN) and Hepatic cytochrome *b*_5_/P450 reductase null (HBRN) mice showed a significant increase in diol levels compared with microsomes of untreated mice [40,41]. Similarly, the presence of the diol was found after BaP incubation of microsomes from *Trp53*(+/+) mice pretreated with BaP, with increased levels in *Trp53*(+/−) and *Trp53*(−/−) mice [11]. BaP metabolism was also studied in F258 rat liver epithelial and mouse hepatoma Hepa1c1c7 cells, where the diol was formed in both, with Hepa1c1c7 having higher levels, but tetrol formation seen only in Hepa1c1c7 [42].

Additionally, varying dG-*N*^2^-BPDE levels formed in all organoid types and donor cultures (Figure 4K–O). Highest adduct levels were seen in pancreatic D39, followed by undifferentiated liver and kidney D21 organoids. Intermediate levels were seen in pancreatic D44, followed by kidney D50, gastric D88 and D95, and differentiated liver organoids. Lowest levels were seen in the colon. As with *CYP1A1* expression and BaP metabolite formation, colon organoids had the lowest levels of DNA adducts and there was a difference of 1.5–2-fold between concentrations. BaP-DNA adduct formation has been reported in many different biological systems, including mice and rats, cell lines and in vitro cell-free systems [4,30,32,40,43,44]. Krais et al. [11] measured DNA adduct levels in different organs of mice treated i.p. with BaP or BPDE. With BaP the liver had higher average adduct levels than the kidney, while treatment with BPDE produced more adducts in the kidney. In that study, colon formed higher levels of dG-*N*^2^-BPDE than liver, kidney and glandular stomach. Here, gastric D95 had the same adduct levels as differentiated liver organoids which correlates with the similar levels of adducts formed in glandular stomach and liver of mice [11]. High adduct levels were formed in Muta^TM^ Mouse liver across different BaP doses, more than double the levels in glandular stomach, comparable to what was seen here in undifferentiated liver and gastric organoids [45]. In a recent study by Long et al. [44] BaP induced the highest level of DNA adducts in liver, followed by kidney and glandular stomach of male Muta^TM^ Mouse, an order comparable to the present organoid study. Similarly, higher adduct levels in the liver compared to glandular stomach and colon were seen in mice administered BaP [6]. In another study [4], DNA adduct levels in mice administered BaP for 1 or 5 days were higher after 5 days of exposure and higher in glandular stomach and kidney compared to colon and liver. Thus, although there are conflicting findings on the levels of BaP-DNA adduct formation in individual tissues, some similarities can be found. No studies have compared adduct levels in pancreas to other tissues, although adducts have been reported to be formed in pancreatic cell systems [7].

Lastly, in order to investigate tissue-specific responses in more depth, an analysis of expression changes of genes involved in different pathways was carried out using HT RT-qPCR. Overall, significant changes were seen across all organoid types and six gene group classifications (Supplementary Figure S1). Similar studies carried out in mice and human cell lines exposed to BaP or BPDE have revealed alterations to phase I and II metabolic enzymes, DNA damage response, pro-apoptotic and oxidative stress response genes [6,16,25]. Additionally, some of these studies compared responses between target and non-target mouse organs and although the modulation of some genes was significantly different, there was still overlap between target and non-target organs [6]. Of the organs studied here it cannot be stated definitively which are target organs for human cancer by BaP and which are non-target due to the absence of any direct epidemiological evidence [1].

The genes with the most significant changes in the XM group were *NQO1* and *UGT1A.* Upregulation of *NQO1* followed a similar pattern to that seen in the analysis of XMEs (Figure 2), and *UGT1A* had the highest upregulation in pancreatic organoids (Figure 5A,B). *NQO1* upregulation has been reported previously and *UGT1A* has been found to be significantly induced by BaP in 3D HepG2 cultures, where *CYP1A1* was also induced but at much higher levels [18]. Additionally, *UGT1A* expression was differentially regulated in MCF-7 cells [34]. DDR genes with most significant changes were *MGMT*, *GADD45A*, *DDB2* and *RRM2B* (Figure 6). In contrast to what was seen here, expression of *MGMT* was upregulated in forestomach and lung of BaP-treated mice [16,46]. Piberger et al. [25] found *GADD45A, DDB2* and *RRM2B* to be induced in human TK6 cells by a range of BPDE concentrations. This is in line with what was seen here and reported in HepG2 spheroids, primary human T lymphocytes, human lung fibroblasts and MCF-7 cells [18,30,37,38,47]. The PCC gene *CDKN1A* was upregulated (Figure 5E), which partly correlates with what was seen in DDR protein expression where p21 was induced in most organoid cultures, and with what has been reported in studies involving different human cells [18,25,30,37,38,43,47] and in BaP-treated mice [16]. The OS, TF and apoptotic genes, *TXNRD1, MDM2* and *BBC3*, respectively, were upregulated after BaP treatment (Figure 5C,D,F). Upregulation of *MDM2* has also been reported in TK6 cells, human primary T lymphocytes and mouse lung and forestomach tissues. In contrast, no change was seen in HepG2 spheroids [16,18,25,38,46]. There was no significant upregulation of *BBC3* mRNA expression in human T lymphocytes; however, ≥2-fold induction was seen in TK6 and MCF-7 cells [25,34,38], while *TXNRD1* was upregulated in HepG2 and MCF-7 cells [30,43].

In conclusion, organoids derived from normal human stomach, colon, pancreas, kidney and liver tissues were all capable of activating BaP, leading to different cytotoxic and genotoxic effects. This was confirmed by the induction of XMEs, DDR proteins, BaP metabolite and BaP-DNA adduct formation. Several changes were observed by HT RT-qPCR analysis. Induction of some of these genes included *CDKN1A*, *MDM2* and *GADD45A* and protein expression of p-p53 and p21 indicating the involvement of the p53 pathway in response to BaP treatment. Of the organs studied here it cannot be stated definitively which are target organs for human cancer by BaP and which are non-target, but it is conceivable that some of the different tissue responses observed determine the tumour specificity of BaP. These initial studies indicate that human tissue organoids are a good system to investigate the cellular responses to carcinogens and its organotropism. Further studies are in progress with agents that have better defined human target tissues in order to provide insights into carcinogen organotropism.

## 4. Materials and Methods

### 4.1. Human Material for Organoid Cultures

Gastric tissue (two donors, designated D88 and D95) was from the Wellcome Trust Sanger Institute, Hinxton, UK, in accordance with the London-Camden and King’s Cross Research Ethics Committee (REC#16/L0/1110). Pancreatic (two donors, D39 and D44) and liver (one donor, D4) tissues were from Addenbrooke’s Hospital, Cambridge, UK, in accordance with the NRES Committee East of England-Cambridge Central (REC#12/EE/0253; 16/EE/0227). Kidney tissue (two donors, D21 and D50) was from the Princess Maxima Centre for Paediatric Oncology, Utrecht, The Netherlands in accordance with the Medical Ethical Committee of the Erasmus Medical Center (Rotterdam, The Netherlands; REC#MEC-2016-739). Colon tissue (two donors, D311 and D351) was from the Department of Paediatrics, University of Cambridge, Cambridge, UK, in accordance with the East of England Cambridge South Research Ethics Committee (REC#17/EE/0265). Further details can be found in Supplementary Table S1.

### 4.2. Organoid Culture

Organoids were grown in 24-well plates, embedded in BME2 gel (Cultrex, Minneapolis, MN, USA; #3533-010-02) or Matrigel (Corning, Corning, NY, USA; #356231), and overlaid with organoid type-specific growth medium (Supplementary Table S2) as described [19,20,21,22,23]. Growth medium was changed every 2–3 days. Organoids were passaged every 7–10 days, depending on density, by mechanical shearing or enzymatic digestion with TrypLE (Gibco, Waltham, MA, USA; #12605028); after passaging organoid media was supplemented with 10 µM of the ρ-associated protein kinase inhibitor Y27632 (Stem Cell Technologies, Vancouver, BC, Canada; #72308). Liver organoid differentiation into hepatocytes was carried out as reported [21].

### 4.3. BaP Treatment

Stock solutions of BaP (Sigma, St Louis, MO, USA; purity ≥ 96%) in dimethylsulfoxide (DMSO) at 25 mM were stored in aliquots at −20 °C until use. Organoids were seeded in 96-well or 24-well plates 48 to 72 h before treatment. Stock solution was diluted in organoid medium to the desired final concentrations and the organoids treated for 48 h. Solvent controls were treated with 0.5% DMSO.

### 4.4. Cell Viability Assessment

Cell viability was measured using the CellTiter-Glo 3D Cell Viability Assay according to the manufacturer’s instructions (Promega, Madison, WI, USA; G9683). The reagent was added after 48 h treatment in a 1:2 ratio with the media. After 30 min incubation at room temperature, 50 µL was transferred to a white assay plate and luminescence was measured using a GloMax Explorer microplate reader (Promega). Each treatment was performed at least in triplicate.

### 4.5. RT-qPCR

Details of RNA sample preparation are provided in Supplementary Materials. qPCR reactions were performed using TaqMan Gene Expression Master Mix (Applied Biosystems, Waltham, MA, USA; #4369016) and Roche Universal Probe Library intron-spanning assays for the following NCBI sequences: NM_000499.3 (*CYP1A1*) and NM_000903.2 (*NQO1*). Each reaction was run at least in triplicate using ABI Fast optical 96-well (Applied Biosystems, #4346907) or 384-well reaction plates on an ABI Prism 7500 or 7900HT Fast Real-Time PCR machine (Applied Biosystems). Relative gene expression was normalised to the housekeeping gene *GAPDH* (NM_002046.5) and analysed by the comparative threshold cycle method (C_t_). Results are reported as the relative fold change in expression (2^−ΔΔCt^) between the treated and solvent control samples.

High-throughput RT-qPCR analysis was carried out with Fluidigm (San Francisco, CA, USA) dynamic arrays on the BioMark^TM^ System as described [25].

### 4.6. Western Blotting

When organoids reached a density of 70–80% they were treated for 48 h with BaP concentrations that resulted in 30%, 50% and 80% viability. Organoids were then harvested and incubated in TryLE for 10 min at 37 °C to remove the membrane matrix. The organoid pellet was washed with cold PBS and lysed in 62.5 mM Tris (pH 6.8), 1 mM EDTA (pH 8.0), 2% sodium dodecyl sulfate, 10% glycerol, 1X HaltTM Protease and Phosphatase Inhibitor Cocktail (Thermo Fisher Scientific, Waltham, MA, USA; #78442). Western blotting was carried out as described [48]. The primary antibodies used were: anti-p21 (1:2000; BD Bioscience, Franklin Lakes, NJ, USA; #BD556431), anti-phospho-H2AX (Ser139, 1:1000; Cell Signalling, Danvers, MA, USA; #9718S), anti-phospho-CHK2 (T68, 1:1000; Cell Signalling, #2197S), anti-phospho-p53 (Ser15, 1:2000; Cell Signalling, #9284S) and anti-GAPDH (1:25,000; Chemicon, Tenecula, CA, USA; #MAB374).

### 4.7. Metabolite Analysis

BaP metabolite analysis by HPLC fluorescence detection was performed essentially as described [49]. Further details are provided as Supplementary Information.

### 4.8. DNA Adduct Analysis by LC-ESI-MS/MS

Organoids were harvested after 48 h treatment and DNA isolated using standard phenol-chloroform extraction. Samples were analysed using liquid chromatography-electrospray ionization tandem mass spectrometry (LC-ESI-MS/MS) at the Masonic Cancer Center, University of Minnesota [50]. The dG-*N*^2^-BPDE adducts were quantified by positive ion targeted MS^2^ (tMS^2^) analysis using a Lumos Orbitrap Tribrid mass spectrometer (Thermo Scientific, Waltham, MA, USA). Experimental details on sample preparation and mass spectrometry analysis are provided in Supplementary Materials.

### 4.9. Statistical Analysis

Results are shown as mean ± SD. Sample size is indicated in each section. *GraphPad Prism* versions 8.4.3 and 9 (GraphPad Software Inc., La Jolla, CA, USA) were used for statistical analyses. Relative mRNA expression data were log2 transformed with a one sample *t*-test with Bonferroni correction against the control mean of 0 (* *p* < 0.05; ** *p* < 0.01; *** *p* < 0.001, difference from control).

## Data Availability

Additional supporting data are available on request to the corresponding author.

## Supplementary Materials

Supporting information can be downloaded at: https://www.mdpi.com/article/10.3390/ijms24010606/s1.
