# Supplementary material for: Metabolic Activation of Benzo[a]pyrene by Human Tissue Organoid Cultures
Source: Int J Mol Sci. 2022 Dec 29;24(1):606. doi: 10.3390/ijms24010606 (PMC9820386; doi:10.3390/ijms24010606)
Supplement: Supplementary file 1 [file ijms-24-00606-s001.zip › Supplementary Figure S1.pdf]

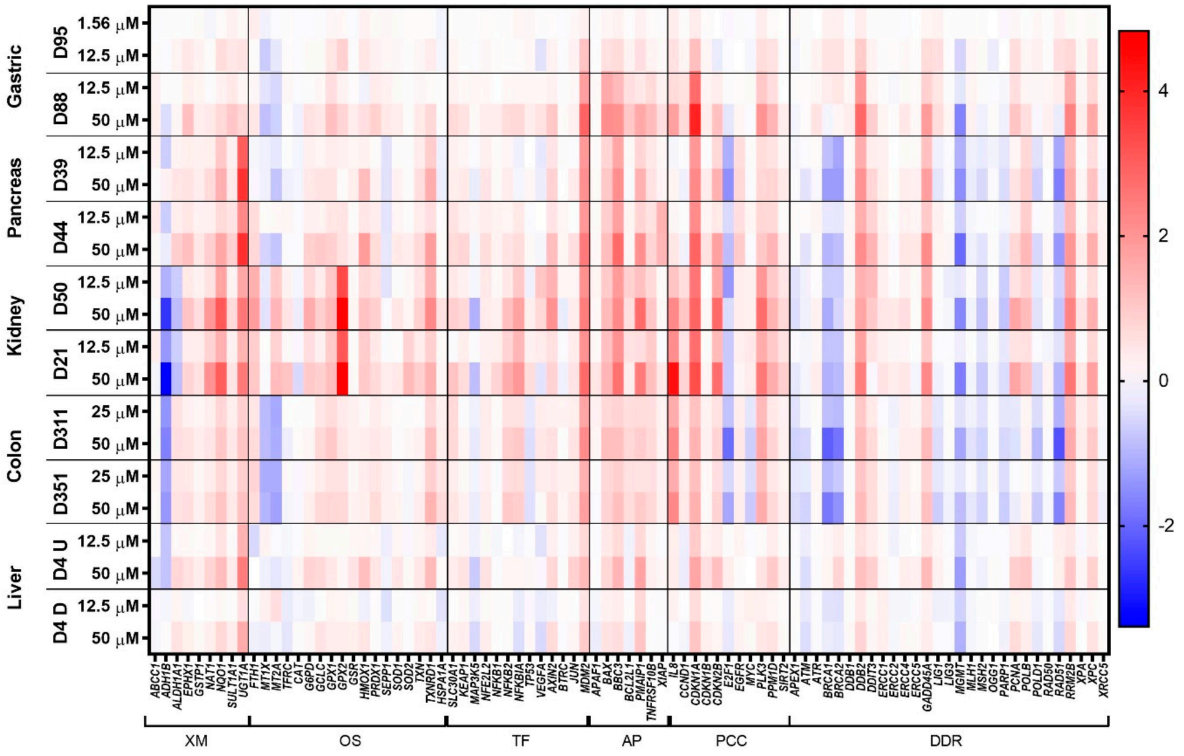

Figure S1. Heatmap of the relative gene expression in human organoids after BaP treatment. HT RT-qPCR was carried out with samples from gastric (D95 and D88), pancreas (D39 and D44), kidney (D50 and D21), colon (D311 and D351) and liver (D4 undifferentiated and differentiated) organoids after treatment with BaP for 48 hr. Upregulated and downregulated genes are shown in red and blue, respectively. Genes that were not analysed are displayed as X. XM: Xenobiotic metabolism; OS: Oxidative stress response; TF: Transcription factors; AP: Apoptosis; PCC: Proliferation and cell cycle control; DDR: DNA damage response and repair. Results shown are log2 transformed and relative to the DMSO control (n=3).
