# Supplementary material for: Metabolic Activation of Benzo[a]pyrene by Human Tissue Organoid Cultures
Source: Int J Mol Sci. 2022 Dec 29;24(1):606. doi: 10.3390/ijms24010606 (PMC9820386; doi:10.3390/ijms24010606)
Supplement: Supplementary file 1 [file ijms-24-00606-s001.zip › Supplementary Tables S1-S3_01092022.pdf]

Table S1. Information about organoid donors from different human tissues

Tissue	Donor ID	Specific biopsy information	Collaborating research team	Research Ethics Committee (REC)	REC #	HMDMC/ HumFre #
Gastric	D88	Normal gastric tissue from the upper stomach was removed from two patients (Donor 88 and Donor 95) that were undergoing biopsy for an oesophageal tumour. Organoid cultures were derived from the tissue at the Sanger Institute as previously described (Li 2018).	Hayley Francies/ Mathew Garnett (Sanger)	London - Camden and Kings Cross Research Ethics Committee	16/L0/1110	18/054
	D95					
Colon	SC311 (D311)	Normal sigmoid colon tissue taken by endoscopy from healthy, control donors (note that this was part of an IBD study).	Matthias Zilbauer (Department of Paediatrics, University of Cambridge)	East of England Cambridge South Research Ethics Committee	17/EE/0265	21/0016
	SC351 (D351)					
Kidney	JD021 (D21)	Normal tissue was taken following nephrectomy or biopsy. (REF: An organoid biobank for childhood kidney cancers that captures disease and tissue heterogeneity; 2020)	Jarno Drost (Princess Maxima Centre for Pediatric Oncology, Utrecht, the Netherlands)	Medical ethical committee of the Erasmus Medical Center (Rotterdam, the Netherlands).	MEC-2016-739	21/0015
	JD050 (D50)					
Pancreas	D39	Biopsies of normal pancreatic or liver tissue were obtained from deceased patients during surgery for organ transplantation.	Meritxell Huch (Max Planck Institute of Molecular Cell Biology and Genetics)/ Kourosh Saeb-Parsy (Department of Surgery, Addenbrookes Hospital)	NRES Committee East of England - Cambridge Central	12/EE/0253; 16/EE/0227	18/071
	D44					
Liver	D4					18/071

Table S2. Growth media recipes for organoids derived from different human tissues

Medium component	Final concentration							Source
	Schlaermann	Pancreas	Liver	Kidney	Colon	Liver Starting	Liver Differentiation	
Advanced DMEM/F12	√	√	√	√	√	√	√	Life technologies, #12634-010
HEPES	10 mM	10 mM	10 mM	10 mM	10 mM	10 mM	10 mM	Life Technologies, #15630-056
Glutamax	1X	1X	1X	1X	1X	1X	1X	Life technologies, #35050-038
WNT3A CM	50%	-	-	-	50%	30%	-	ATCC CRL-2647 cell line
R-Spondin-1 CM	25%	10%	10%	10%	20%	10%	-	Cultrex, #3710-001-01
N-acetyl cysteine	1.25 mM	1.25 mM	1.25 mM	1 mM	1.25 mM	1.25 mM	1.25 mM	Sigma, #A9165
Human Noggin	150 ng/mL	25 ng/mL	-	-	-	25 ng/mL	-	Peprotech, #120-10C
Mouse Noggin	-	-	-	-	100 ng/mL	-	-	Peprotech, #250-38-250ug
Recombinant Human EGF	20 ng/mL	50 ng/mL	50 ng/mL	50 ng/mL	-	50 ng/mL	50 ng/mL	Gibco, #PHG0313
Recombinant Mouse EGF	-	-	-	-	50 ng/mL			Gibco, #PMG8041
FGF-10	150 ng/mL	100 ng/mL	100 ng/mL	100 ng/mL	-	100 ng/mL	-	Peprotech, #100-26-25
HGF	-	-	25 ng/mL	-	-	25 ng/mL	25 ng/mL	Peprotech, #100-39
Gastrin I human	10 nM	10 nM	10 nM	-	10 nM	10 nM	10 nM	Sigma, #G9020
FGF-19	-	-	-	-	-	-	100 ng/mL	R&D, #969-FG-025
B27 Supplement (+ Vitamin A)	1X	-	-	1.50%	1X	-	1X	Invitrogen, #17504001
B27 Supplement (- Vitamin A)	-	1X	1X	-	-	1X	-	Invitrogen, #12587001
A83-01	1 μM	5 μM	5 μM	5 μM	0.5 μM	5 μM	0.5 μM	Tocris, #2939
PGE2	-	3 μM	-	-	-	-	-	Tocris, #2296
N2 Supplement	1X	1X	1X	-	-	1X	1X	Gibco, #17502001
Forskolin	-	10 μM	10 μM	-	-	10 μM	-	Tocris, #1099
Nicotinamide	10 mM	10 mM	10 mM	-	10 mM	10 mM	-	Sigma, #N0636
SB202190	2 μM	-	-	-	10 μM	-	-	Stem Cell Technologies, #72634
DAPT	-	-	-	-	-	-	10 μM	Sigma, #D5942

Medium component	Final concentration							Source
	Schlaermann	Pancreas	Liver	Kidney	Colon	Liver Starting	Liver Differentiation	
Dexamethasone	-	-	-	-	-	-	3 µM	Sigma, #D4902
BMP7	-	-	-	-	-	-	25 ng/mL	Peprotech, #OP1 120-03
hES Cell Cloning and Recovery	-	-	-	-	-	1X	-	Stemgent, #01-0014-500

Table S3. Genes identified as biologically relevant in at least three organoid types after HT RT-qPCR analysis

				Gene Classification																	
				Xenobiotic metabolism		Oxidative stress response	Transcription factors	Apoptosis			Proliferation and cell cycle control				DNA damage response and repair						
				NQO1	***UGT1A***	***TXNRD1***	***MDM2***	***PMAIP1***	***BAX***	***BBC3***	***CDKN1A***	***CDKN2B***	***IL8***	***PLK3***	***BRCA1***	***DDB2***	***GADD45A***	***MGMT***	***RRM2B***	***XPC***
**Average log2-fold ratio of BaP-treated samples to vehicle control (BaP [µM])**	Stomach	D88	**12.5**	0.1954	0.1109	0.0668	1.8081	0.4064	1.5964	1.2767	2.7463	0.3204	0.6491	1.0188	0.3776	1.9346	0.8413	-0.3333	1.5188	0.7310
			**50**	0.6975	0.7433	0.7190	2.9285	1.6151	2.1393	2.0772	4.0546	1.1509	1.7934	2.0144	-0.1391	2.8355	1.8918	-1.6464	2.3348	1.6096
		D95	**1.56**	0.0650	0.0793	0.0222	0.2590	0.2369	0.1380	0.1959	0.3681	-0.0008	0.3172	0.3450	0.2100	0.3417	0.1654	0.0477	0.0829	0.0831
			**12.5**	0.6171	0.5085	0.3664	1.1179	0.5993	0.5565	0.6560	1.2007	-0.2583	0.5505	0.6182	0.5037	0.9507	0.6479	-0.5632	0.7152	0.5531
	Pancreas	D44	**12.5**	0.6749	2.2367	0.5952	1.9156	0.7579	0.9065	1.7614	2.1599	0.3902	0.1287	0.7528	-0.2945	1.5017	0.9838	-0.6160	1.2767	0.6689
			**50**	1.6698	3.8430	1.8294	2.4969	2.0918	1.2295	2.7522	2.9155	1.7688	0.4499	1.0070	-0.9679	2.1531	2.1940	-1.9658	1.9655	1.5219
		D39	**12.5**	0.9859	3.0186	0.9398	1.9035	0.7592	0.7680	1.6830	1.9245	0.7057	-0.0950	0.6432	-0.8505	1.5440	1.1394	-1.0585	1.2977	0.6032
			**50**	1.5104	3.8031	1.5510	2.0703	1.4753	1.0173	2.0862	2.2105	1.4575	0.1829	0.7543	-1.3242	2.0619	1.8033	-1.5218	1.6114	1.1281
	Liver	D4 Und.	**12.5**	0.5052	1.4298	0.3759	0.7229	0.6970	0.3863	0.6511	0.9631	0.3519	0.2919	0.0597	0.1309	0.5581	0.4044	-0.6940	0.4218	0.2379
			**50**	1.2151	2.4602	1.2826	1.2888	1.7066	0.7195	1.3660	1.6892	1.1711	0.3058	0.4803	0.1610	1.1608	1.2959	-1.3105	1.0362	0.8392
		D4 Diff.	**12.5**	0.1107	0.5825	0.0725	0.4916	0.7239	0.2229	0.3288	0.6074	0.0264	0.1942	0.2643	-0.3718	0.4305	0.2076	-0.6019	0.3856	0.1106
			**50**	0.7195	1.5815	0.4717	0.9602	1.1142	0.5028	0.5818	1.0763	0.1660	-0.0274	0.4475	-0.2611	0.7856	0.3577	-1.0616	0.7295	0.5379
	Kidney	D50	**12.5**	1.7180	1.6468	1.4455	2.0680	1.1573	0.9544	1.8907	2.5837	1.6348	1.0841	1.9191	-0.6292	1.4419	1.4544	-0.2874	1.7112	0.6360
			**50**	3.1033	2.5711	2.2397	2.4257	2.7607	1.2659	2.1887	2.8660	2.8197	2.2176	2.7293	-1.1169	1.6931	2.1243	-1.5874	2.3221	1.5109
		D21	**12.5**	1.4791	1.4132	0.8611	2.0467	0.5983	1.1559	2.0626	2.7150	1.3012	2.1161	1.5611	-0.8030	1.7584	0.9925	-0.2191	1.5220	0.7216
			**50**	3.0202	2.5109	1.4868	2.7795	2.4745	1.3636	2.6361	3.3010	2.7446	4.3890	2.5338	-1.0518	2.4143	2.2375	-1.7270	2.5928	1.7610
	Colon	D311	**25**	0.7400	0.8895	0.7096	1.6908	0.6284	0.7048	0.7963	1.1208	0.5175	1.5690	1.2815	-0.8472	1.0182	0.7101	-0.3672	1.2911	0.6157
			**50**	1.0302	1.0813	1.2215	2.1740	0.9385	1.2044	1.1924	1.4331	0.7611	2.2522	1.6916	-2.1199	1.2714	1.2842	-1.1890	1.6062	1.0023
		D351	**25**	0.7232	0.9257	0.8630	1.4591	0.5465	0.7188	0.8728	1.1829	0.5887	1.5499	1.1955	-0.9431	1.0530	0.8153	-0.2987	0.9556	0.6112
			**50**	1.1014	1.1407	1.4017	1.7968	0.9474	0.7864	1.1372	1.4766	0.7751	2.2392	1.5600	-1.7755	1.2543	1.1617	-0.8674	1.2854	0.7091
